# A brief history of anaesthesia in South Africa

**DOI:** 10.4102/jcmsa.v1i1.53

**Published:** 2023-12-22

**Authors:** Peter C. Gordon, Michael F. James

**Affiliations:** 1 Department of Anesthesia and Perioperative Medicine, Faculty of Health Sciences, University of Cape Town, Cape Town, South Africa

News of the first public demonstration of the use of ether to annul the pain of surgery by William Morton in October 1846, spread rapidly around the world. In April 1847, ether was being used in Cape Town. The first published use of ether for major surgery in South Africa took place in Grahamstown in June 1847 when Dr Guybon Atherstone administered ether anaesthesia for a below knee amputation of a leg of the Deputy Sheriff for Albany, Mr F. Carlisle. The Sheriff was reported to have said:

… [*T*]he greatest boon ever conferred on mankind, I have been totally unconscious of everything – the sound of that horrid saw still grates as in a dream … but as for pain I have felt not the slightest.^1^

## Early anaesthetists

During the Anglo–Boer War, anaesthesia was used by both sides. British medical services stored chloroform and ether at every base hospital. Mounted doctors carried chloroform in their saddlebags.^2^

Dr George Warwick Bampfylde Daniell came out to SA as a doctor in the Anglo-Boer War bringing with him a state-of-the-art Clover inhaler. He settled in SA and became SA’s first ‘specialist anaesthetist’ when he was appointed as Anaesthetist to the Johannesburg Hospital in 1907. He later moved to Cape Town and became the first lecturer in anaesthesia to University of Cape Town (UCT) medical students at the New Somerset Hospital. The majority of anaesthetics were given by general practitioners (GPs) in private homes using ‘rag and bottle’ techniques with ether or chloroform delivered through a Schimmelbusch mask.

Anaesthetists in private practice at that time were not paid for work in State Hospitals but were paid a fee for services by the surgeon they worked for in private practice. Anaesthetists also had to carry all their equipment except oxygen to private hospitals.

The anaesthetic challenges during World War I led to advances including the development of Boyles apparatus and endotracheal intubation. Despite this, concern over the high rate of anaesthetic fatalities resulted in the Medical Association of SA (1927) declaring that it was ‘… unprofessional to employ an unregistered person as a paid anaesthetist’.

In the 1930s, several SA anaesthetists travelled to the United States (US) to learn new techniques. These included Johannesburg’s Benjamin Weinbren and Cape Town’s Royden Muir who, in 1933, became the first South African to present at a Congress in the US.

## Specialisation

In 1935, the Royal College of Surgeons in England recognised anaesthesia as specialty and introduced the Conjoint DA (London) as a higher qualification. Several SA anaesthetists travelled overseas to obtain the qualification. In 1953, the Royal College of Surgeons introduced the Fellowship of the Faculty of Anaesthetists of the Royal College of Surgeons (FFARCS), a precedent South Africa later followed.

The US equivalent was Board Certification. The first US board certified South African, C.S. ‘Buck’ Jones (1935), returned to SA and was appointed Head of Department at UCT and Groote Schuur Hospital in 1952.^3^

Ongoing concern over deaths from anaesthesia led to a government enquiry (1936). This is reported as follows:

- Anaesthesia attributable deaths 0.93/1000
- Chloroform deaths 1:560
- Ether deaths 1:3166

This frighteningly high fatality rate led to a government stipulation that training facilities were to: ‘ensure that medical students and resident medical officers have every opportunity of acquiring adequate practical experience in administering anaesthesia’.

It was further stipulated that it was ‘desirable that in hospitals … trained anaesthetists should be employed, and as part of their duty should teach residents’. This recommendation stimulated better training for anaesthetists.

In 1938, South African Medical and Dental Council of South Africa (SAMDC) recognised and registered Medical Specialties. The SAMDC decreed that the administration of anaesthetics was restricted to medical practitioners although dentists were also allowed to administer anaesthetics.

## South African Society of Anaesthetists

**FIGURE 1:**
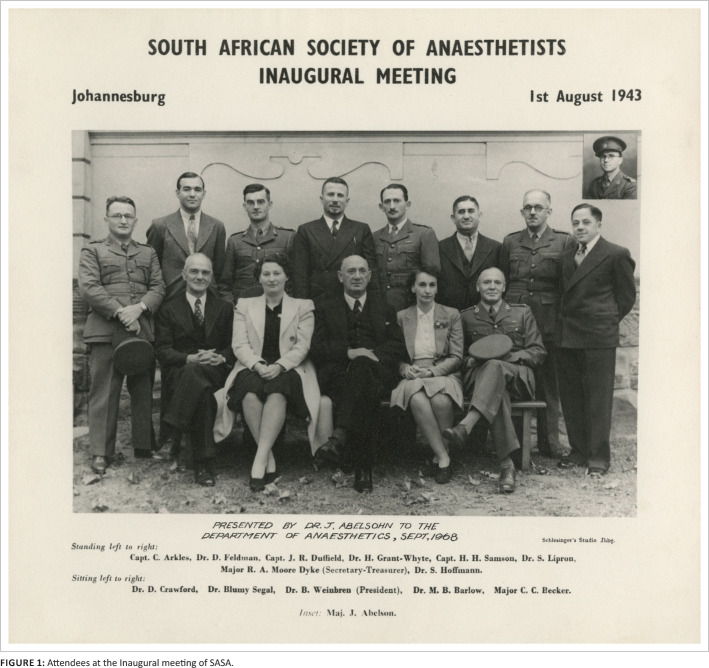
Attendees at the Inaugural meeting of SASA.

When war broke out in 1939, anaesthetists were required on the battlefield, giving females a chance to enter a male dominated specialty. During the war, a meeting was held at the Johannesburg Hospital in August 1943 at which South African Society of Anaesthetists (SASA) was formed (see Figure 1). At that time, there were only 26 anaesthetists (including three women) on the SAMDC Register. Dr Benjamin Weinbren was elected as President. The aims of the Society were:

[*T*]o promote the science of anaesthesia, to determine the relationships that should exist between anaesthetists, and between anaesthetists and hospitals, public and private institutions, government and the medical profession and to represent and further the interests of anaesthetists.^4^

South African Society of Anaesthesiologists has evolved into a highly professional society serving both the anaesthetic community and the general public. It has become one of the leading voices and influencers in the health sector in South Africa.^4^ The society has published numerous guidelines to practice that have had a major impact on anaesthetic safety.

## Chairs of anaesthesia

At the 34th SA Medical Congress in 1946, anaesthesia was given an entire session to commemorate the Centenary of Morton’s ether demonstration. At the SAMA AGM a surgeon, Mr Aubrey Radford, proposed ‘the immediate instigation of a Chair of Anaesthesia for SA’. This eventuated in 1959 when O.V.S. Kok was appointed to the Chair of Anaesthesia at Pretoria University. Subsequently, chairs were created at the universities of The Witwatersrand (1962), Natal (1964), Cape Town 1965, Stellenbosch (1970), Free State (1971) and Medunsa (1978).^5^

The establishment of university chairs in anaesthesia led to a rapid expansion of anaesthesia research in South Africa. Foremost among these researchers was Prof. Gaisford G. Harrison whose seminal study into anaesthetic mortality over a 30-year period remains the most substantial mortality study in the field.^6^ Harrison also performed unique research into a new, anaesthetic-related condition known as malignant hyperthermia. He introduced dantrolene as the solution to this problem and this publication was selected by *British Journal of Anaesthesia* (BJA) in its centenary year (2023) as one of a selected series of classic papers in anaesthesia.^7^

Anaesthesia, and particularly Dr Joseph Ozinsky, played a major role in the success of the world’s first heart transplant in 1967.^8,9^

## The World Federation of Societies of Anaesthetists

World Federation of Societies of Anaesthetists (WFSA) was formed in Holland in 1955. South Africa was a founder member and was represented at the meeting by Dr Roberts. In 1960, Prof. Kok became the first South African elected to the WFSA Executive Committee

At the First All Africa Congress held in Harare in 1997, the opening address was delivered by Prof. Harrison and the African Region of WFSA was established.

## The College of Anaesthetists

The College of Physicians and Surgeons of South Africa (SA) (Colleges of Medicine of SA) was established in 1954. Dr Francis W Roberts proposed the establishment of a Faculty of Anaesthetists at the first AGM of the CMSA in 1956. The SAMDC recognised the FFA(SA) as a specialist qualification in 1957.^10^ The first Diploma in Anaesthesia was awarded by the Faculty in 1963. A full College of Anaesthesia within CMSA was established in 1998.

In 1980, SA, represented by Harrison, together with the UK, Ireland, Australia and NZ, Canada and the American Board of Anesthesiology, was a founder member of the Conference of International Reciprocating & Examining Boards of Anaesthesia (CIREBA). The SA hosted CIREBA in 1988.

The College of Anaesthetists of the Colleges of Medicine of SA was formed in 1995 and Prof. Mike James was elected as the first president.^10^ Prof. David Morrell became the first anaesthesiologist elected President of the Colleges of Medicine in 1998.

## The 2008 World Congress Cape Town

In 2008, South Africa hosted the 8th World Congress of Anaesthesiologists at the Cape Town International Convention Centre and 6224 delegates attended. The Organising Committee was chaired by Prof. Dave Morrell and the Scientific Programme organised by Profs Mike James and Andre Coetzee. Financially and scientifically it was a great success. The surplus was dedicated to SASA’s education initiatives.

## Conclusion

Anaesthesia has a proud history of training, practice and research in this country that is recognised as world class and has made a proud contribution to the development of medicine in South Africa.
